# Development of a Trivalent Construct Omp18/AhpC/FlgH Multi Epitope Peptide Vaccine Against *Campylobacter jejuni*

**DOI:** 10.3389/fmicb.2021.773697

**Published:** 2022-01-13

**Authors:** Hongqiang Lou, Xusheng Li, Xiusheng Sheng, Shuiqin Fang, Shaoye Wan, Aihua Sun, Haohao Chen

**Affiliations:** ^1^Medical Molecular Biology Laboratory, School of Medicine, Jinhua Polytechnic, Jinhua, China; ^2^Shanghai Prajna Biotech Co., Ltd., Shanghai, China; ^3^Department of Pathogen Biology and Immunology, School of Basic Medicine and Forensic Medicine, Hangzhou Medical College, Hangzhou, China

**Keywords:** *Campylobacter jejuni*, outer membrane proteins, T-B combined antigenic epitope, immunogenicity, Omp18/AhpC/FlgH

## Abstract

*Campylobacter jejuni* (*C. jejuni*) is one of the major pathogens contributing to the enteritis in humans. Infection can lead to numerous complications, including but not limited to Guillain-Barre syndrome, reactive arthritis, and Reiter’s syndrome. Over the past two decades, joint efforts have been made toward developing a proper strategy of limiting the transmission of *C. jejuni* to humans. Nevertheless, except for biosecurity measures, no available vaccine has been developed so far. Judging from the research findings, Omp18, AhpC outer membrane protein, and FlgH flagellin subunits of *C. jejuni* could be adopted as surface protein antigens of *C. jejuni* for screening dominant epitope thanks to their strong antigenicity, expression of varying strains, and conservative sequence. In this study, bioinformatics technology was adopted to analyze the T-B antigenic epitopes of Omp18, AhpC, and FlgH in *C. jejuni* strain NCTC11168. Both ELISA and Western Blot methods were adopted to screen the dominant T-B combined epitope. GGS (GGCGGTAGC) sequence was adopted to connect the dominant T-B combined epitope peptides and to construct the prokaryotic expression system of tandem repeats of antigenic epitope peptides. The mouse infection model was adopted to assess the immunoprotective effect imposed by the trivalent T-B combined with antigen epitope peptide based on Omp18/AhpC/FlgH. In this study, a tandem epitope AhpC-2/Omp18-1/FlgH-1 was developed, which was composed of three epitopes and could effectively enhance the stability and antigenicity of the epitope while preserving its structure. The immunization of BALB/c mice with a tandem epitope could induce protective immunity accompanied by the generation of IgG2a antibody response through the *in vitro* synthesis of IFN-γ cytokines. Judging from the results of immune protection experiments, the colonization of *C. jejuni* declined to a significant extent, and it was expected that AhpC-2/Omp18-1/FlgH-1 could be adopted as a candidate antigen for genetic engineering vaccine of *C. jejuni* MAP.

## Introduction

*C. jejuni* is a Gram-negative, spiral-shaped bacterium that is deemed to be a commensal inhabitant of gastrointestinal tracts of chickens and wild birds (Hermans et al., 2012). Consumption of contaminated poultry meat is a critical source of clinical infection and has emerged as the leading factor contributing to the foodborne bacterial gastroenteritis in humans (European Food Safety Authority and European Centre for Disease Prevention and Control (EFSA and ECDC), 2018). In addition, *C. jejuni* constitutes an invasive microorganism that could cause gastroenteritis associated with fever and frequent watery bloody diarrhea, abdominal pains, and occasional nausea (Silva et al., 2011; Bintsis, 2017). It is also linked with post-infection complications such as the immune-mediated neurological disorders of the Guillain-Barré Syndrome (GBS) (Nachamkin, 2002; Alshekhlee et al., 2008; Stojanov et al., 2020), its variant Miller Fisher Syndrome (MFS), or reactive arthritis (Altekruse et al., 1999; Ang et al., 2001). Notably, the infectious dose is thought to be lower than the one for other foodborne pathogens given that merely 500–800 bacteria could trigger human infection (Castano-Rodríguez et al., 2017).

Such a microaerophilic, capnophilic, and thermophilic microorganism requires fastidious growth conditions and its growth can be promptly hampered by several conditions of environmental stress. Optimal growth can be obtained by adopting a modified atmosphere limited in dioxygen and enriched in carbon dioxide, in addition to the temperature of (37^°^C, 45^°^C) and the pH value of (6.5, 7.5) (Macé et al., 2015). However, *C. jejuni* is able to survive harmful conditions by forming adaptation mechanisms in response to stress conditions throughout the food chain (Rodrigues et al., 2016). Living in a biofilm is also regarded as a phenotypical feature demonstrated by *C. jejuni*, indicating numerous survival methods outside hosts (Turonova et al., 2015). There has been no standard method for exploring the biological roles of *C. jejuni*. Moreover, the *in vitro* cell invasion or chicken and mouse models of intestinal colonization are adopted in the majority of these studies. In fact, chicken is the natural reservoir of *C. jejuni*, and another animal model is C57BL/6J IL-/- mice, which serves as an imperfect model of human enteritis (Cayrou et al., 2021).

Vaccination is an effective means of preventing infectious diseases. Judging from the data set out in the epidemiologic and human challenge studies, biological feasibility of vaccine development can be supported. Passive immunization could lower the possibility of colonization and transmission of *C. jejuni* in broiler chickens (Hermans et al., 2014). So far, there has been no vaccine approved by any global regulatory authority targeted at preventing Campylobacter-associated diseases (Liu et al., 2019). Nevertheless, several efforts are underway to design vaccines against *C. jejuni*, including the conventional approach of adopting the live attenuated or heat-killed whole cells as potential vaccines. In addition, several subunit vaccines, and particularly the adoption of bacterial outer membrane protein (Omp), alkyl hydroperoxide reductase (AhpC), and Flagellar L-ring protein precursor (FlgH), have proved to have bright prospects in terms of lowering the bacterial load in experimental hosts (Meunier et al., 2016). Recombinant flagellar proteins such as FlaA, FliG, FliE, and FlgH could have potential for novel targets for vaccine development (Yeh et al., 2013). However, the genetic engineering vaccine with single protein antigen features an optimal immune effect on virus infection with simple surface protein antigen, but could only impose limited protective effect on bacterial infection with complex surface protein antigen (Jelínková et al., 2021). Epitope multi-antigen peptide vaccine is a newly developed genetic engineering vaccine, which is artificially cross-linked by multiple dominant epitopes identified by reverse vaccinology and macromolecules core carrier. Such vaccine features the advantages of simultaneous presentation of multiple antigenic epitopes, greater presentation of antigen, and non-covalent binding between antigenic peptide branches so as to form epitopes and to enhance immune effects. In the meantime, individual amino acid mutations could not affect presentation of antigen and the subsequent immune response (Lozano et al., 2021). Over the recent years, it is found that *C. jejuni* Omp18, AhpC outer membrane protein, and FlgH flagellin subunit feature numerous advantages, including but not limited to strong antigenicity, expression in varying strains, and sequence conservation (Watson et al., 2014; Guérin et al., 2020; Quintel et al., 2020). Therefore, Omp18, AhpC, and FlgH could be adopted as surface protein antigens for screening dominant epitopes of *C. jejuni*. In this study, we have explored the preparation of epitope multi-antigen peptide vaccine against the aforementioned outer membrane proteins and assess their respective effect, so as to lay the foundation for the development of *C. jejuni* polyantigen peptide and genetically engineered vaccine.

## Materials and Methods

### Prediction of T-B Binding Epitopes in Outer Membrane Proteins Omp18, AhpC, and FlgH of *Campylobacter jejuni*

The complete sequence of the genome of *C. jejuni* strain NCTC11168 was obtained from the National Center for Biotechnology Information.^1^ The sequences of Omp18, AhpC, FlgH genes and their products were extracted from GenBank with the accession numbers of NC_002163.1, CAL34485.1, and RNF61095.1, respectively. The signal peptides and sequences of Omp18, AhpC, and FlgH were predicted through the use of SignalP 4.1^2^; the transmembrane domain was predicted through the use of TMHMM^3^; the B-cell epitopes were predicted through the use of the online software IEDB^4^; and the T-cell epitopes were predicted through the online algorithm for identifying the ligation strength.^5^ Based on the score of T-cell epitope and B-cell epitope as well as their coincidence, the combined epitope of T-B was predicted accordingly.

### Strains, Plasmids, and Epitope Peptides

*C. jejuni* NCTC11168 strain was purchased from the National Collection of Type Cultures (NCTC); Escherichia coli DH5 α, BL21DE3 and their competent bacteria were purchased from Shanghai Weidi Biotechnology Co., Ltd.; PET30a plasmids were purchased from Invitrogen Corporation based in the United States; the Omp18, AhpC, and FlgH genes were synthesized by Detai Biotechnology (Nanjing) Co., Ltd.; and the pET30a plasmid vector was established accordingly. The restriction sites of Nde I and Hind III were sequenced by Shanghai Introvigen Co. Moreover, Omp18, AhpC, FlgH epitope peptides, and T- and B-combined epitope peptides were established by Suzhou Qiangyao Biological Technology Co., Ltd. through the use of the technology of solid phase peptide synthesis, the raw material of N-α-Fmoc protected amino acid, the carrier of Fmoc-AA-Wang resin, and methodology of HBTU activation. As can be found through the HPLC test, the purity exceeded 95%.

### Antibodies and Kits

HRP-labeled goat anti-rabbit IgG (SA00001-3, 1:5,000) was purchased from Proteintech Group; the fetal bovine serum (Catalog No: 04-001-1A) was purchased from the Israeli company of Biological Industries; the Bradford protein concentration assay kit (Catalog No: P0013) was purchased from Shanghai Beyotime Biotechnology Co., Ltd.; and the Mouse IFN- γ (Catalog No: 70-EK280/3-96) and IL-4 (Catalog No: 70-EK204/2-96) ELISA kits were purchased from Multisciences Biotech Co.

### Preparation of Antisera Against Omp18, AhpC, and FlgH

SPF female BALB/c mice were purchased from Shanghai SLAC Laboratory Animal Co., Ltd., and male New Zealand rabbits were purchased from Shanghai Jiesijie Laboratory Animal Co., Ltd. Six healthy male New Zealand rabbits, each weighing about 3 kg, were selected for the experiments. This research project was approved by the Medical Ethics Committee of Jinhua Polytechnic (No. JHCDW2018012, Jinhua, China). Preparation of antisera in rabbits followed the same procedures as specified in our previous study (Lou et al., 2019). About 2 mL of blood was obtained from the ear marginal vein of rabbits before immunization. The separated serum was mixed with normal saline containing 60% glycerol at 1:1 as the negative control. Subsequently, the epitope peptides of rOmp18, rAhpC, or rFlgH (1 mL, 2 mg) were fully emulsified with BCG, with an equal volume of Freund’s incomplete adjuvant (1 mL), and the resulting mixture was adopted to inject New Zealand rabbits for the generation of antiserum.

Immunity procedure: rabbits were immunized with 2 mL of emulsified antigen (1 mg/mL) on both sides of the back spine via subcutaneous injection. Immunization was conducted once a week (three times in total). Rabbits were anesthetized with the use of pentobarbital (30 mg/kg, intravenous). Moreover, the blood was collected from the heart 10 days subsequent to the final immunization. Serum was separated from the collected blood and IgG in the serum was enriched by saturated ammonium sulfate precipitation and DEAE-52 column chromatography. The supernatant was extracted and stored at −80^°^C for reserve.

### Determination of Antibody Titer by ELISA

Briefly, ELISA plates were coated with 5 μg/mL of each of the rOmp18, rAhpC, and rFlgH recombinant proteins in assay buffer (PBS with 1% BSA and 0.05% Tween 20) for 1 h at 37^°^C. After blocking and washing, antiserum diluted 1:5,000 in assay buffer was added to ELISA plates, and were incubated for 1 h at 37^°^C. The control group was rabbit serum prior to immunization, which was incubated at 37^°^C for 1 h and rinsed for three times. The substrate chromogenic solution was added to each well away from light for 5 min, and the reaction was terminated by rinsing with distilled water. In addition, the value was read at 450 nm, and the rabbit serum with the highest antibody titer was extracted for the follow-up Dot Blot detection and the ELISA detection for dominant epitopes.

### Detection of Dominant Epitopes by Dot Blot and ELISA

For dot blot studies, epitope peptides samples were diluted to the desired concentrations in Tris-buffered saline (TBS) and loaded on 0.4 mm PVDF membranes and blocked with 5% milk. The membrane was washed with Tris-buffered saline with Tween (TBST) buffer, incubated with primary antibodies at room temperature for 2 h, washed three times with TBST for 10 min each time, exposed in a darkroom, developed, and immersed in SuperSignal™ West Dura Extended Duration substrate (Thermo Fisher Scientific, Waltham, MA, United States), and exposed to X-ray film in the later stage. The detection method of epitopes was briefly as follows: diluted the sample and placed it in the plate, by adding the rOmp18-IgG, rAhpC-IgG, and rFlgH-IgG antibody, then the enzyme-labeled second antibody was added, substrate solution for the color reaction, and finally, terminated the reaction by adding the stop solution. Last but not least, the value was measured by the enzyme labeling instrument.

### Establishment of the rAhpC-2/Omp18-1/FlgH-1 Recombinant Expression Vector

AhpC-2, Omp18-1, and FlgH-1 peptide sequences were connected with the GGS sequence (GGCGGTAGC) to establish the T-B combined epitope peptide triple repeat concatenation gene. Based on the MaxCodon™ Optimization Program (V13), the amino acid codons were optimized, and gene fragments were synthesized through the method of total gene synthesis. The target gene was inserted into the prokaryotic expression vector pET30a through restriction nuclease cut sites Nde I and Hind III. Subsequent to the double enzyme digestion and sequencing to verify the correct sequence of the recombinant expression vector, the target gene was converted into *E. coli* DH5a and BL21DE3, respectively.

### Immune Procedure, Strain Culture, Identification, and Preparation of Bacterial Suspensions

Ninety SPF female BALB/c mice aged between 4 and 6 weeks were randomly divided into the following five groups, namely, Group A (25 μg rAhpC-2/Omp18-1/FlgH-1 plus complete Freund’s adjuvant); Group B (25 μg rAhpC plus complete Freund’s adjuvant); Group C (25 μg rOmp18 plus complete Freund’s adjuvant); Group D (25 μg rFlgH plus complete Freund’s adjuvant); and Group E (equal amount of normal saline plus complete Freund’s adjuvant, which served as the control group). Mice were given subcutaneous injection on a weekly basis and received four times in total.

Laboratory frozen *C. jejuni* strains were incubated under standard laboratory microaerobic conditions (i.e., 42°C, 10% CO_2_, 5% O_2_, and 85% N_2_) for 24 h. After passing to the third generation, the strains were identified using matrix assisted laser desorption ionization-time of flight mass spectrometry (MALDI-TOF-MS). The identified strains were prepared as *C. jejuni* suspensions, whereas the colony forming units (CFUs) were calculated through the method of plate inoculation. The turbidity of the bacterial solution was modified to 5 × 10^9^ CFU/mL.

### Isolation and Culture of Spleen Lymphocytes

The cells of spleen lymphocytes were isolated from the spleen tissues of BALB/c mice and cultured for 10 days in RPMI-1640 medium supplemented with 10% Fetal Bovine Serum, 1% L-glutamine, 5 U/mL penicillin/streptomycin, 1 mM sodium pyruvate, 50 μg/mL gentamicin, 10 mM HEPES, and 10% non-essential amino acids (Rahe et al., 2020). Moreover, the spleen lymphocytes were calculated via the cell counting plate. In addition, 10^5^ cells were inoculated in the cell culture plate, whereas 2 μL ConA (5 μg/mL) and 50 μL *C. jejuni* lysate (whole cell sediment was resuspended in PBS solution, and in the meantime, the bacterial lysate was obtained by sonication) were added. Moreover, a blank control group was established. Subsequently, the cells were incubated at 37°C for 24 h,, and the culture was centrifuged at 3,000 r/min for 10 min. Then, the supernatant was extracted and stored temporarily at −20°C for the later ELISA detection of IFN- γ and IL-4 levels.

### Detection of Splenic Lymphocyte Subpopulations by Flow Cytometry

In a nutshell, the experimental procedures are specified as follows: 100 μL of cell suspension was added per tube with 50 μL each of anti-mouse PE-CD4, FITC-CD8, APC-CD3, IL4-IgG, and IFN-γ-IgG antibodies. Moreover, equal amounts of PE-IgG, FITC-IgG, and APC-IgG were added to the isotype control tube. Then, the tube was incubated for 30 min at room temperature away from light. Flow cytometry was adopted to measure the intracellular cytokines interferon (IFN)- γ and interleukin (IL)-4 in the spleen lymphocytes so as to identify the Th1 cells (IFN-γ (+) IL-4 (+) CD4 (+) cells), Th2 cells (IFN-γ (−) IL-4(+) CD4(+) cells), Tc1 cells (IFN-γ+ IL-4 (−) CD8 (+) cells), and Tc2 cells (IFN-γ (−) IL-4(+) CD8(+) cells).

### Detection of IFN- γ and IL-4 Levels by ELISA

The suspensions from each cultured well were collected to examine the concentration of IFN-γ and IL-4 with the ELISA kits based on the manufacturer’s instructions. A total of 100 μL suspensions were added to sample wells, whereas 50 μL diluted detection antibody (1:100) were added to each well and then incubated at 300 r/min for 2 h at room temperature. After 6 times of rinsing with the 300 μL wash solution, 100 μL of horseradish peroxidase-labeled streptavidin (1:100) were added and then incubated at 300 r/min for 45 min at room temperature. After 6 times of rinsing with the 300 μL wash solution, 100 μL of TMB were added and then incubated for 5–30 min at room temperature. The STOP Solution was added to terminate the reaction, and the absorbance was immediately measured using a microplate reader at 450 nm.

### Animal Protection Experiment

BALB/c mice were randomly divided into five groups, namely, the rAhpC-2/Omp18-1/FlgH-1 group, the rOmp18 group, the rAhpC group, the rFlgH group, and the normal control group, with 13 mice allocated into each group. Each mouse in the first four groups was subcutaneously injected with 25 μg corresponding recombinant protein and the Freund’s complete adjuvant on a weekly basis for 4 times in total. The oral *C. jejuni* (1 mL, 5 × 10^9^ CFU) treatment was given on days 1, 3, and 7 subsequent to the final immunization. Next, the morbidity and death of animals were examined on a daily basis within 8 weeks, whereas the disease index of each group was calculated accordingly. The scoring criteria was specified as follows: 0 refers to physical health, 1 refers to symptoms and signs, and 2 refers to death. Mice have shown one or more signs and symptoms including the significant reduction of activity, loss of appetite, shrugging of the back, loose and gray fur, and depression. The disease index of each group was divided by the total number of animals observed on the same day. Subsequently, the rate of immune protection was calculated based on the following formula: protection rate = (disease index of the control group - disease index of the immune group)/disease index of the control group × 100%. The intestinal tissue of mice was stained using HE and examined through the ordinary light microscope. For the intestinal mucosa that did not suffer from an injury but showed mild inflammatory cell infiltration, such symptom was categorized as mild inflammation. For the intestinal mucosa that accumulated moderate inflammatory exudate where partial necrosis was discovered, such symptom was categorized as moderate inflammation. For the intestinal mucosa that was nearly completely exfoliated with the pathologic features of a large number of inflammatory cell infiltration, such symptoms of inflammatory exudate and necrosis were categorized as severe inflammation.

### Data Processing and Statistical Analysis

The experimental data were expressed as mean ± standard deviation ($\bar{\text{x}}$ ± SD), whereas the multi-group mean analysis was conducted with the statistical analysis software of GraphPad Prism. First, the experimental data were examined for homogeneity of variance. In case the variance was homogeneous, then the one-way ANOVA was adopted for overall comparison. The means of each experimental group and the control group were statistically analyzed through the LSD method, and the data of non-normal or uneven variance were statistically analyzed through the rank sum test.

## Research Findings

### Sequence Retrieval and Epitope Analysis of Outer Membrane Protein of *Campylobacter jejuni*

Based on previous studies (Gupta et al., 2017) and for the purpose of achieving the optimal immunogenicity, we have selected Omp18, AhpC, and FlgH of *C. jejuni* NCTC11168 strains as the epitope-based vaccines to predict the B-cell epitope and T-cell epitope. Based on the scores of the T-cell and B-cell epitope as well as their coincidence (i.e., the T-B combined epitope), we have obtained six high-score T-B combined epitopes (as specified in Table 1). Further analysis can be conducted on this basis so as to identify the vaccine strategy for *C. jejuni* strain NCTC11168.

**TABLE 1:** High scores of OMP18, AhpC, and FlgH of *Campylobacter jejuni* T-B combined antigenic epitopes.

MAP	Position	MAP sequence
AhpC-1	110–132	ARNF***DVLVAEAVALRGSFLL***DAD
AhpC-2	4–39	TKKALDFTAPAVLGNNEIVQDFNLYKNIGP***KGAVVF***
Omp18-1	117–158	KAVKE***ALIAKGVNADRIAVK***SYGETNPVCTEKTKACDAQNRR
Omp18-2	3–47	KIL***FTSIAALAVVISGCS***TKSTSVSGDSSVDSNRGSGGSDGWDID
FlgH-1	14–52	*FGCSATVDPQISMKP* PAYVEELAPKQSNNVESAPGSLFG
FlgH-2	64–97	AMNVNDLVTVVIQ*ESTTQSTQANKATSRTNTDSL*

*The underline represents the B epitope, the square represents the human T epitope, and the bold italic represents the mouse T epitope.*

### Expression, Purification, Immunogenicity Detection, and Combined Epitope Screening of a Target Recombinant Protein of *Campylobacter jejuni*

**FIGURE 1:**
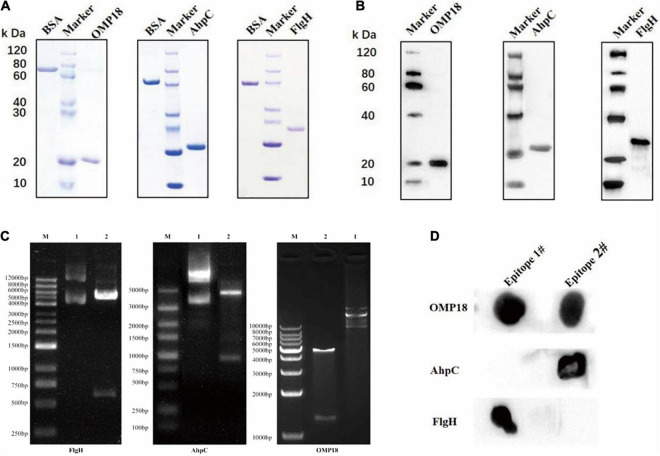
Expression, purification, immunogenicity detection, and combined epitope screening of a target recombinant protein of *Campylobacter jejuni*. **(A)** SDS-PAGE results showed that the rOmp18 (∼17 kDa), rAhpC (∼22 kDa), and rFlgH (∼24 kDa) purified by Ni-NTA affinity chromatography. **(B)** Western Blot results showed that rOmp18, rAhpC, and rFlgH could specifically immunobind to the corresponding antibodies. **(C)** The Nde I and Hind III double enzyme digestion results were located correctly after the *Campylobacter jejuni* Omp18, AhpC, and FlgH genes were ligated to pET30a. **(D)** Dot Blot assay showed the binding ability of antigen and antibody.

The results of Nde I and Hind III double enzyme digestion were properly located after the *C. jejuni* Omp18, AhpC, and FlgH genes were located to pET30a (as illustrated in Figure 1C). The established prokaryotic expression systems of Omp18, AhpC, and FlgH genes of *C. jejuni* were able to effectively express the target recombinant proteins of rOmp18, rAhpC, and rFlgH under the induction of IPTG, SDS-PAGE, and the results of the rOmp18 (∼17 kDa), rAhpC (∼22 kDa), and rFlgH (∼24 kDa) purified by Ni-NTA affinity chromatography were all shown as single protein bands (as illustrated in Figure 1A). According to the Bradford method, the concentrations of the purified rOmp18, rAhpC, and FlgH were measured to be 0.745, 1.150, and 0.381 mg/mL, respectively. In the meantime, the Western Blot results showed that rOmp18, rAhpC, and rFlgH were able to specifically immunobind to the corresponding antibodies (as illustrated in Figure 1B). The results of Dot Blot assay showed that among the two T-B combined epitope of *C. jejuni* Omp18, AhpC, and FlgH, rOmp18-IgG could bind to the epitope of Omp18-1 and Omp18-2. Nevertheless, the binding reaction of the former was stronger than that of the latter, whereas rAhpC-IgG AhpC and rFlgH-IgG only presented strong binding results to AhpC-2 and FlgH-1, respectively (as illustrated in Figure 1D), indicating that Omp18-1, AhpC-2, and FlgH-1 were the dominant T-B combined epitope.

### Identification of *Campylobacter jejuni* AhpC-2/Omp18-1/FlgH-2 Tandem-Epitope Peptide

**FIGURE 2:**
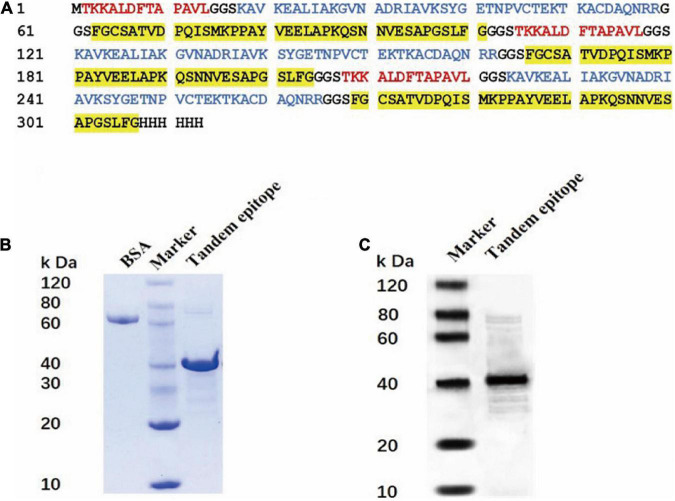
Construction and protein identification of tandem epitope of Rahpc-2/OMP18-1/Flgh-1. **(A)** Amino acid sequence of tandem epitope peptide of rAhpc-2/OMP18-1/Flgh-1. The red, blue, and yellow background fonts represent different epitopes. Red: Ahpc-2 epitopes, blue: OMP18-1 epitopes, yellow: Flgh-1 epitopes. SDS-PAGE **(B)** and Western Blot **(C)** analysis of the tandem epitope of rAhpc-2/OMP18-1/Flgh-1.

The rAhpC-2/Omp18-1/FlgH-1 recombinant expression vector was established and connected to a dominant T-B combined epitope peptide using a GGS sequence in the tandem epitope amino acid sequence. Moreover, the prokaryotic expression system of a repeated tandem epitope peptide gene was established accordingly (as illustrated in Figure 2A), in which the SDS-PAGE detection results showed that IPTG could induce the expression of the trivalent recombinant T-B combined epitope peptide rAhpC-2/Omp18-1/FlgH-1 in a tandem repetitive prokaryotic expression system (as illustrated in Figure 2B). In addition, the Western Blot results showed that rAhpC-2/Omp18-1/FlgH-1 could lead to a strong specific immune binding reaction with the whole-bacterium antibody of *C. jejuni* (as illustrated in Figure 2C).

### Tandem Epitopes Can Significantly Increase the IgG2a and IFN-γ Levels of Splenic Lymphocyte *in vitro*

**FIGURE 3:**
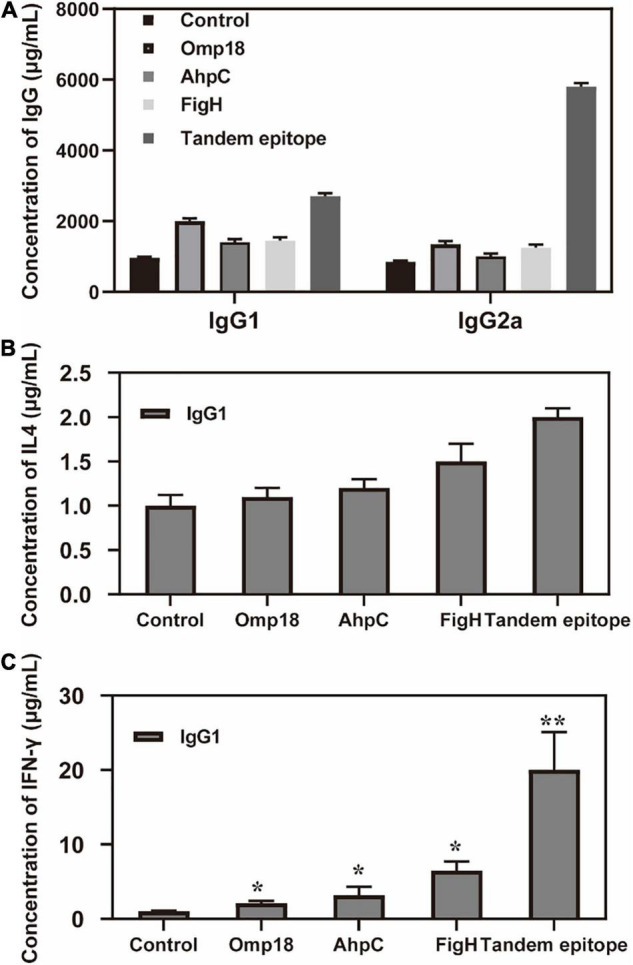
Tandem epitopes significantly increase the IgG2a and IFN-γ levels of splenic lymphocyte *in vitro*. **(A)** Changes of IgG level in mice immunized with different epitopes. **(B)** Changes of IL-4 level in spleen Lymphopoiesis of mice immunized with different epitopes. **(C)** Changes of IFN-γ level in spleen Lymphopoiesis of mice immunized with different epitopes. *P < 0.05, **P < 0.01, compared with control group.

**FIGURE 4:**
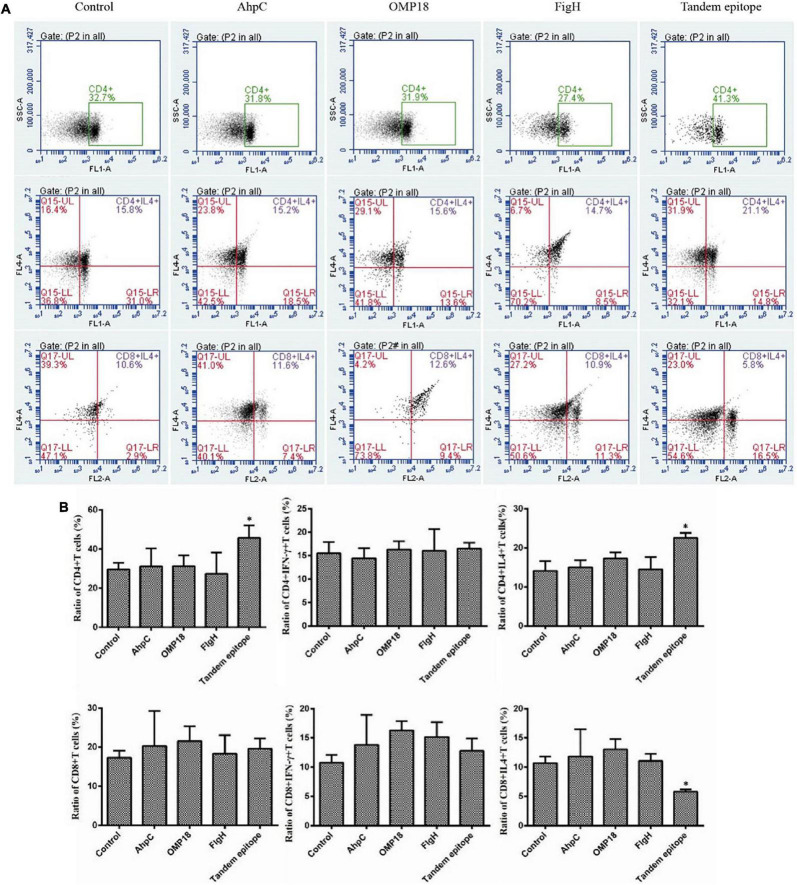
Tandem epitopes significantly increased the number of Th1 cells and decreased the number of Tc2 cells *in vitro*. **(A,B)** Flow cytometry was used to measure the intracellular cytokines interferon (IFN)-γ and interleukin (IL)-4 in the spleen lymphocytes so as to identify the Th1 cells [IFN-γ(+) IL-4(+) CD4(+) cells] Th2 cells [IFN-γ(-) IL-4(+) CD4(+) cells], Tc1 cells [IFN-γ+ IL-4(-) CD8(+) cells], and Tc2 cells [IFN-γ(-) IL-4(+) CD8(+) cells)]. *Compared with control group (*P* < 0.05).

Judging from the ELISA results, the serum levels of the IgG antibody of mice immunized with rAhpC, rOmp18, and rFlgH all went up (*P* < 0.05). However, the increase in serum IgG of mice immunized with T-B combined with rAhpC-2/Omp18-1/FlgH-1 tandem epitope peptide was found to be more significant (*P* < 0.05), and the major types included IgG1 and IgG2a (as shown in Figure 3A). Judging from the ELISA results, the IFN-γ levels in the supernatant of spleen leucocyte-immunized mice immunized with rAhpC, rOmp18, rFlgH, and rAhpC-2/Omp18-1/FlgH-1 tandem epitope peptides were all up-regulated. In the meantime, the increase was found to be the most significant in IFN-γ in the mice immunized with rAhpC-2/Omp18-1/FlgH-1 tandem epitope peptide (*P* < 0.05), but there was no significant variation in the IL-4 level in the supernatant (*P* > 0.05) (as shown in Figures 3B,C, respectively). Judging from the results of flow cytometry, no major changes were found in the number of CD4+ cells in spleen lymphocytes of mice immunized with rOmp18, rAhpC, and rFlgH (*P* > 0.05), but the number of CD4+ cells experienced significant increases in the spleen lymphocytes of mice immunized with the tandem epitope peptide of rAhpC-2/Omp18-1/FlgH-1 (*P* < 0.05). In particular, the number of CD4+ IL-4+ cells went up to a significant extent (*P* < 0.05), but the number of CD4+IFN-γ+ cells experienced no major variations (*P* > 0.05). Moreover, no major changes were found in rAhpC, rOmp18, rFlgH, and the number of CD8+ cells as well as the number of IFN-γ+ CD8+ cells in the spleen lymphocytes of mice immunized with the rAhpC-2/Omp18-1/FlgH-1 tandem epitope peptide (*P* > 0.05). However, the number of CD8+IL-4+ cells experienced significant declines in the mice immunized with the rAhpC-2/Omp18-1/FlgH-1 tandem epitope peptide (*P* < 0.05) (as shown in Figures 4A,B).

### Tandem Epitopes Can Significantly Inhibit the *Campylobacter jejuni* Colonization in the Intestine and Intestinal Mucosal Inflammation in Mice

**FIGURE 5:**
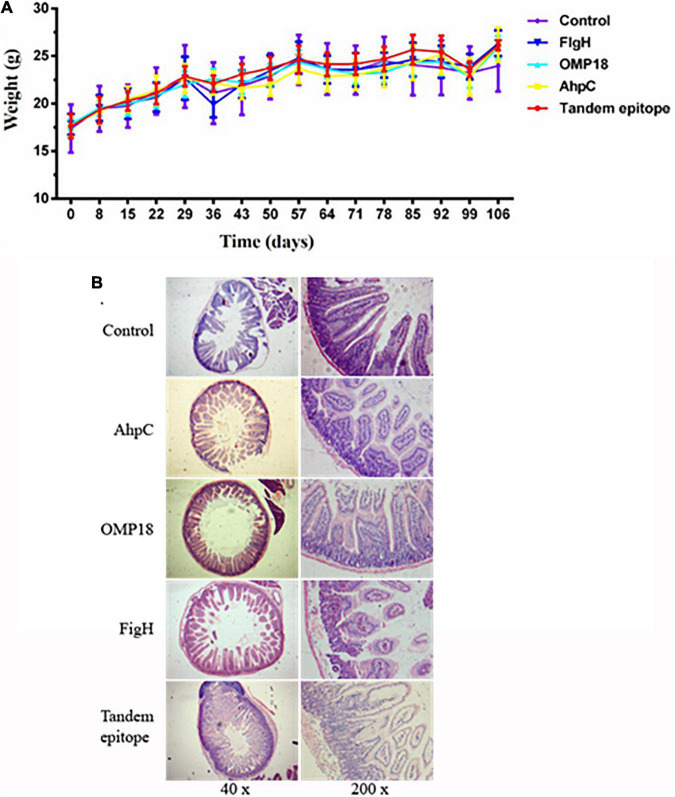
Tandem epitopes significantly reduced *Campylobacter jejuni* colonization in the intestine and intestinal mucosal inflammation in mice. **(A)** Weight change in mice immunized with different epitopes. **(B)** Pathological changes of intestinal mucosa in mice immunized with different epitopes.

Judging from the animal experiments, after the mice in the non-immunized control group were infected with *C. jejuni* through the digestive tract, the positive rate of *C. jejuni* in the jejunum contents amounted to 90%, whereas the number of colonies could reach 10^4^ CFUs. The positive rate of *C. jejuni* all experienced significant reductions in the jejunum contents of mice immunized with rOmp18, rAhpC, rFlgH, rAhpC-2/Omp18-1/FlgH-1 tandem T-B, and epitope peptide (*P* < 0.05). In addition, the positive rate of *C. jejuni* in mice immunized with rAhpC-2/Omp18-1/FlgH-1 declined by 40%, which was significantly lower than that in mice immunized with rOmp18, rAhpC, and rFlgH (*P* < 0.05). As shown by the analytical results, the disease index of mice immunized with rAhpC-2/Omp18-1/FlgH-1 was significantly lower than that of mice immunized with rOmp18, rAhpC, and rFlgH-1 (*P* < 0.05), whereas the protection rate could reach up to 80% (as specified in Table 2). Subsequent to the immunization, the body weights of mice in each group increased without major variation (as illustrated in Figure 5A). Judging from the pathological examinations, more inflammatory cell infiltration was found in the intestinal mucosa of the rOmp18, rAhpC, and rFlgH-immunized mice, whereas the intestinal mucosa of the rAhpC-2/Omp18-1/FlgH-1-immunized mice remained intact and showed no signs of major inflammatory cell infiltration (as illustrated in Figure 5B).

**TABLE 2:** Disease index and protection rate of immune mice infected with *Campylobacter jejuni*.

Groups	Disease index	Protection rate %
rAhpC-2/OMP18-1/FlgH-1	0.20 ± 0.42*^#^	80
rAhpC	0.50 ± 0.71*	50
rOMP18	0.60 ± 0.52*	40
rFlgH	0.70 ± 0.67*	30
Negative control	1.0 ± 0.47	10

*Compared with control group (P < 0.05); ^#^compared with rOmp18, rAhpC, and rFlgH immune groups (P < 0.05).

## Discussion

*C. jejuni* is a major burden to public health in both socioeconomically developing and industrialized nations, which can lead to acute enteritis in humans mainly via the infection of food through the digestive tract (Maziero and de Oliveira, 2010). The incidence of infectious diarrhea caused by *C. jejuni* ranks as the top among all diseases in certain countries and regions, and is second only to Shigella or Salmonella in some other countries. More importantly, *C. jejuni* infection is closely linked with the refractory autoimmune diseases such as GBS, reactive arthritis, and Reiter’s syndrome (Talukder et al., 2011). Therefore, it is of utmost significance to prevent and control the infection caused by *C. jejuni*.

Although varying studies provided evidence of acquired immunity following exposure to *C. jejuni*, effective vaccines were still yet to be developed. Hence, it is an imperative task to identify potential vaccine candidates for Campylobacter species (Motamed-Gorji et al., 2017). The whole-cell vaccine of *C. jejuni* was faced with huge limitations due to the close relationship between *C. jejuni* and the pathogenesis of GBS (Lin et al., 2011). Therefore, it is of utmost practical significance to develop a genetically engineered vaccine that is novel, efficient, and safe against *C. jejuni*. Over the recent years, the MAP vaccine has become a hotspot issue during the research and development of the third generation genetic engineering vaccine against pathogenic microorganisms thanks to its numerous advantages, including but not limited to the ability to simultaneously present multiple antigen epitopes; strong antigen presentation; establishment of constitutional epitopes after non-covalent binding between each antigen-peptide branch; enhanced immune effect; and the fact that individual amino acid mutations will not affect antigen presentation and subsequent immune response (Neal-McKinney et al., 2014; Hisham and Ashhab, 2018; Galen et al., 2021). However, there have been no reports on the MAP vaccine against *C. jejuni* so far.

Protective protein antigen is regarded as a prerequisite for developing the genetically engineered vaccine. An ideal candidate of antigen is required to be surface exposed, recognized by the immune system, widely distributed, capable of preventing infection of both the same and varying strains, and conservative in sequence to obtain stable immune protective effect (Burnens et al., 1995; Wu et al., 2020). With respect to *C. jejuni*, Omp18 is highly conserved in the *C. jejuni* strains isolated from humans, dogs, cats, calves, and chicken, but has shown discrepant features in other Campylobacter species. Moreover, Omp18 belonging to the family of PALs is well conserved in *C. jejuni* and is highly immunogenic. Hence, it is an ideal candidate as the antigen for the serological diagnosis of past *C. jejuni* infections (Lou et al., 2019). In our previous studies, we have screened and identified the AhpC B cell dominant epitope of *C. jejuni*. Based on our research findings, one epitope was able to recognize the antibodies of AhpC and featured strong antigenicity (Yeh et al., 2013). In addition, previous studies have reported cloning, expression, and purification of *C. jejuni* flagellar proteins in a bacterial expression system. Twelve recombinant proteins were purified including FlgE1, FlgG, FlgK, FliE, FlgH/FliH, and FlaA. Experiments were carried out on the purified recombinant proteins to examine whether they were immunogenic with the use of antibodies from several sources. The BacTrace anti-Campylobacter species antibody reacted only to the FlaA recombinant protein instead of other types of proteins. Moreover, rabbit anti-MOMP2 peptide antibody reacted strongly to FlaA, FliG, FliE, FlhF, FlgG, FlgE1, and FliD recombinant proteins. The aforementioned studies on the antibody indicate that these recombinant flagellar proteins have huge potential for novel targets of vaccine development (Rana et al., 2018). In addition, it is expected that these recombinant proteins can provide us with an extremely useful instrument for examining the host immune response to *C. jejuni*. Therefore, we have opted for the *C. jejuni* Omp18, AhpC, and FlgH for the preliminary study on the MAP vaccine.

The MAP vaccine is composed of the epitope peptide and the high-molecular artificial polymer carrier. The critical step is to screen and obtain the dominant epitope. During the production of antibodies such as IgG, high-titer IgG, these antibodies can only be produced with the assistance of T-cells. Unlike viruses, bacterial surface antigens feature intricate and varying structures. As a result, oftentimes it is hard for the genetically engineered vaccines with a single protein antigen to achieve the optimal effects of immune protection. The reverse vaccinology provides a new strategy of vaccine development in the early twenty-first century (Fimlaid et al., 2014). At the core of the strategy is to obtain vaccine effective antigens in a swift and reliable manner through genome and proteomics prediction, and the reverse screening is adopted for the antibody. In addition, although it is a vaccine applicable to humans, the results of animal protection experiments must be provided during its development, whereas mice are regarded as the most commonly used experimental animals.

Therefore, in this study, we have adopted the biological information technology to predict the T-B combined epitope in human and mouse Omp18, AhpC, and FlgH, and screened the dominant T-B combined epitope by ELISA and Western Blot. Subsequently, the artificial tandem epitope of AhpC-2/Omp18-1/FlgH-1 was established before being recombined and expressed in success, so that the yield was further improved. Moreover, the antigenicity and stability were effectively enhanced due to the fact that the recombined epitope peptide featured a larger molecular weight.

Based on the research findings, intestinal epithelial cells of INT407, peripheral blood mononuclear cells, and CD4+T cells could produce and secrete IFN-γ under circumstances of *C. jejuni* infection (Heimesaat et al., 2020), and IFN-γ played a vital role in regulating the adaptive immune response of *C. jejuni* in the body (Huizinga et al., 2015). Dendritic cells were the cells capable of initiating adaptive immune response in the body. In particular, DC cells played a more critical role in the mucosal adaptive immune response. As reported in the study, after co-culture of *C. jejuni* with DC cells, CD4+T cells were induced to differentiate into IFN-γ+ cells, which took part in the adaptive immune response of *C. jejuni* (Dasti et al., 2010). Our ELISA results also indicated that the IFN-γ level was significantly increased in the spleen lymphocyte supernatant of mice infected with *C. jejuni* via intestinal tract (*P* < 0.05). Furthermore, our flow cytometry assay indicated that the T-lymphocyte CD4+ cells and CD4+IFN-γ+ T-lymphocytes were significantly increased in mice immunized with AhpC-2/Omp18-1/FlgH-1 tandem T-B and epitope peptide (*P* < 0.05). IFN-γ secreted by Th1 cells could facilitate the production of opsonized antibody IgG2a by B cells, thus further enhancing the phagocytosis and killing of pathogens by macrophages, whereas IL-4 secreted by Th2 cells could facilitate the proliferation and differentiation of B-cells into plasma cells, thus generating antibodies mainly composed of IgG1. Therefore, judging from the increase in IFN-γ and the production of high-titer IgG2a in mice immunized with AhpC-2/Omp18-1/FlgH-1 tandem T-B and epitope peptide, both the T and B epitopes in the T-B combined epitope peptide could effectively play their respective roles.

Although the pathogenic mechanism of *C. jejuni* was yet to be fully understood, adhesion and colonization were closely linked with its pathogenesis (Efimochkina et al., 2020; Simson et al., 2020). Mice were the most commonly infected animals with *C. jejuni*. Based on our mouse immunoprotection assay, subsequent to the immunization with AhpC-2/Omp18-1/FlgH-1 tandem T-B and epitope peptides, the colonization of *C. jejuni* was significantly reduced in the jejunum of mice (*P* < 0.05), and the pathological damage to the intestinal mucosa was also significantly alleviated (*P* < 0.05). In summary, AhpC-2/Omp18-1/FlgH-1 could be adopted as the candidate antigens for the MAP genetically engineered vaccine against *C. jejuni*.

## Data Availability Statement

The original contributions presented in the study are included in the article/supplementary material, further inquiries can be directed to the corresponding author/s.

## Ethics Statement

The animal study was reviewed and approved by the Medical Ethics Committee of Jinhua Polytechnic.

## Author Contributions

HL, AS, and HC contributed to conception and design of the study. HL organized the database and wrote the first draft of the manuscript. SW and SF performed the statistical analysis. XL, XS, HL, and HC wrote sections of the manuscript. All authors contributed to manuscript revision, read, and approved the submitted version.

## Conflict of Interest

SF and SW were employed by Shanghai Prajna Biotech Co., Ltd. The remaining authors declare that the research was conducted in the absence of any commercial or financial relationships that could be construed as a potential conflict of interest.

## Publisher’s Note

All claims expressed in this article are solely those of the authors and do not necessarily represent those of their affiliated organizations, or those of the publisher, the editors and the reviewers. Any product that may be evaluated in this article, or claim that may be made by its manufacturer, is not guaranteed or endorsed by the publisher.

## Footnotes

1
http://www.ncbi.nlm.nih.gov/nuccore/AL111168.1

2
http://www.cbs.dtu.dk/services/SignalP/

3
http://www.cbs.dtu.dk/services/TMHMM/

4
http://tools.iedb.org/bcell/

5
http://www.syfpeithi.de/bin/MHCServer.dll/EpitopePrediction.htm
